# Integrative analysis of the metabolome and transcriptome reveals the mechanism of polyphenol biosynthesis in *Taraxacum mongolicum*

**DOI:** 10.3389/fpls.2024.1418585

**Published:** 2024-08-16

**Authors:** Xing Zhao, Yiguo Li, Yuanchong Huang, Jun Shen, Huini Xu, Kunzhi Li

**Affiliations:** ^1^ Faculty of Life Science and Technology, Kunming University of Science and Technology, Kunming, China; ^2^ Plateau Characteristic Agricultural Development Office, Kunming Bureau of Agriculture and Rural Affairs, Kunming, China

**Keywords:** dandelion, polyphenol, flavonoid, chicoric acid, metabolome and transcriptome

## Abstract

**Introduction:**

Dandelion is widely used in clinical practice due to its beneficial effects. Polyphenolic compounds are considered the main anti-inflammatory active ingredient of dandelion, but the gene expression patterns of polyphenolic compounds in different dandelion tissues are still unclear.

**Methods:**

In this study, we combined a nontargeted metabolome, PacBio Iso-seq transcriptome, and Illumina RNA-seq transcriptome to investigate the relationship between polyphenols and gene expression in roots, flowers, and leaves of flowering dandelion plants.

**Results:**

Eighty-eight flavonoids and twenty-five phenolic acids were identified, and 64 candidate genes involved in flavonoid biosynthesis and 63 candidate genes involved in chicoric acid biosynthesis were identified. Most flavonoid and chicoric acid-related genes demonstrated the highest content in flowers. RNA-seq analysis revealed that genes involved in polyphenol biosynthesis pathways, such as CHS, CHI, F3H, F3’H, FLS, HQT, and CAS, which are crucial for the accumulation of flavonoids and chicoric acid, were upregulated in flowers.

**Discussion:**

The combination of transcriptomic and metabolomic data can help us better understand the biosynthetic pathways of polyphenols in dandelion. These results provide abundant genetic resources for further studying the regulatory mechanism of dandelion polyphenol biosynthesis.

## 1 Introduction

Dandelion (*Taraxacum mongolicum* Hand.-Mazz.) is a plant species belonging to the *Taraxacum* genus of the Asteraceae (Compositae) family, with more than 2500 species in the genus (Stork, 1920). It grows in temperate regions around the world, including on lawns, roadsides, fields, and riverbanks, as well as in other areas with moist soil (Fan et al., 2023). Dandelion is a traditional Chinese medicinal herb that has the effects of clearing heat, detoxifying, reducing swelling, dispersing nodules, diuresis and relieving gonorrhea (Fan et al., 2023). It is commonly used in clinical practice to treat inflammation, stomach diseases, tumors, gynecological diseases, and other medical conditions (Schütz et al., 2006; Asadi-Samani et al., 2015). In addition, dandelion, as a high-quality nutritional and health-promoting vegetable, is increasingly favored by people (Di Napoli and Zucchetti, 2021). Dandelion has been recognized as a common herbal medicine and food additive (Sharifi-Rad et al., 2018; Ge et al., 2021). The significant biological functions of dandelion can be attributed to its phytochemical components, including flavonoids, phenolic acids, terpenoids, sterols, volatile oils, polysaccharides, coumarins, and other active compounds (González-Castejón et al., 2012; Martinez et al., 2015; Lis et al., 2020; Liu et al., 2022). Among them, flavonoids and phenolic acids are the main active metabolites of dandelion. Therefore, the biosynthesis and regulation of these active ingredients have received widespread attention.

Flavonoids are a class of secondary metabolites present in medicinal plants that exhibit chemical and functional diversity (Tohge et al., 2016; Liu et al., 2023b). Flavonoids, including luteolin (the most abundant), diosgenin, quercetin, apigenin, kaempferol, rutin and diosmetin, are one of the main functional components of dandelion (Shi et al., 2008a). The proportion of flavonoids in dandelions is approximately 1.35%, and flavonoids are the main source of pharmacological activity in dandelions to a certain extent (Shi et al., 2008a, 2008b; Zhang et al., 2022). Flavonoids are synthesized in three stages, with the first stage converting phenylalanine to coumaroyl CoA, involving enzymes such as phenylalanine ammonia-lyase (PAL), cinnamate 4-hydroxylase (C4H), and 4-coumarate CoA ligase (4CL). The second stage involves the synthesis of flavonols, flavones, and flavanones, and several enzymes are involved in this process, including chalcone synthase (CHS), chalcone isomerase (CHI), flavanone 3-hydroxylase (F3H), flavonoid 3′-hydroxylase (F3′H), flavonoid 3′5′-hydroxylase (F3′5′H), flavonol synthase (FLS), and flavone synthase (FNS). The third stage of anthocyanin synthesis involves dihydroflavonol 4-reductase (DFR), anthocyanidin synthase (ANS), and other enzymes (Tanaka and Ohmiya, 2008; Hichri et al., 2011; Guodong et al., 2019; Hua et al., 2023). Eighteen genes, including 4CL, C4H, CHI, F3H, and FLS, have been identified as key structural genes for the biosynthesis of kaempferol and anthocyanins in *Aster tataricus* (Jia et al., 2023).

The polyphenols in dandelion contain not only flavonoids but also phenolic acids (Hu and Kitts, 2005). Many potentially active phenolic compounds, including chicoric acid, 5/4/3-O-caffeoylquinic acid, caffeic acid, ferulic acid, gallic acid, syringic acid, p-hydroxybenzoic, hydroxyphenylacetic acid, scopoletin, esculetin, esculin, and cichoriin, have been isolated from the *Taraxacum* genus (Schütz et al., 2005; Chen et al., 2011; Mo et al., 2017). The biological activities of phenolic acids and flavonoids are immunostimulatory and antioxidant, respectively (González-Castejón et al., 2012). Phenolic acids and flavonoids both originate from p-coumaroyl-CoA synthesized from phenylalanine, which includes three key enzymes, PAL, C4H, and 4CL. Genome-wide prediction and gene expression analysis revealed that three PALs, one C4H, and five 4CL are involved in the biosynthesis of polyphenols (Lin et al., 2018; Li et al., 2021). According to Chinese Pharmacopoeia (2020), the index component of dandelion is chicoric acid (C_22_H_18_O_12_). The genes involved in the biosynthesis of chicoric acid mainly include PAL, C4H, 4CL, HCT/HQT/HTT (BAHD acyltransferase family) and CAS synthase genes (vacuolar SCPL serine carboxypeptidase-like proteins) (Fu et al., 2021; Liu et al., 2023a). The identification of genes related to the biosynthesis of chicoric acid will lay a solid foundation for further molecular engineering research on dandelion.

To date, most research on the biosynthesis of dandelion polyphenols has focused on the use of second-generation sequencing (SGS) platforms (Liu et al., 2023a). However, second-generation transcriptional data have limitations, including short reads and incorrect splicing tendencies. Third-generation sequencing can compensate for the shortcomings of second-generation sequencing (Chen et al., 2018). Using third-generation transcriptome sequencing methods, information on novel genes has been obtained for many plants, such as *Camellia oleifera*, *Coptis deltoidei*, *Olea europaea*, and *Litchi chinensis* (Guodong et al., 2019; Gong et al., 2020; Zhong et al., 2020; Zhou et al., 2020). The combination of transcriptomics and metabolomics research can explore the correlation between metabolites and genes, thereby revealing changes in phenotype expression (Mesnage et al., 2018). The integration of transcriptomics and metabolomics promotes the identification of plant gene functions and the elucidation of metabolic pathways (Luo et al., 2021). In dandelion, the content of luteolin in roots, leaves, and flowers was determined using high-performance liquid chromatography (HPLC) technology. RNA-seq analysis has shown that F3’H may play a crucial role in the biosynthesis of luteolin (Li et al., 2021). The molecular mechanism controlling the differences in polyphenol content in dandelion among different tissues (roots, leaves and flowers) has not yet been explored.

To identify the key genes responsible for the high polyphenol content and to understand the molecular mechanisms of flavonoid and phenolic acid biosynthesis in dandelion, this study used full-length transcriptome sequencing, RNA-seq, and nontargeted metabolomics techniques to characterize and analyze the metabolic profiles and differentially expressed genes of these tissues. All genes involved in flavonoid and phenolic acid biosynthesis were screened, and their expression patterns in different tissues were studied. These results are helpful for inferring and evaluating the role of encoded proteins in dandelion gene regulation.

## 2 Materials and methods

### 2.1 Plant materials

Dandelion seeds were collected from Dongchuan (26°08′N, 103°18′E), Kunming City, Yunnan Province. After sowing, dandelions with red petioles were selected. After three generations of bagging pollination, a stable and hereditary red petiole dandelion was obtained and named Dongchuan Red for subsequent experiments in this study. The dandelions were grown at the experimental base of the Faculty of Life Science and Technology at Kunming University of Science and Technology; dandelions with similar growth statuses and free of pests and diseases were selected for sampling. During the flowering period, leaves (L), flowers (F), and roots (R) were collected separately from each dandelion plant. The materials were flash frozen in liquid nitrogen and stored at −80°C until further use.

### 2.2 Metabolites extraction

Nontargeted metabolite analysis was performed by Novogene (https://cn.novogene.com/). For nontargeted LC−MS/MS analysis, six biological replicates were used for the tissue samples. Dandelion tissues (100 mg) were individually ground with liquid nitrogen and transferred to a 2 mL centrifuge tube, followed by the addition of 500 μL of prechilled 80% methanol. The samples were incubated on ice for 5 min. Then, the supernatants were separated from the mixture by centrifugation at 15,000 × g for 20 min at 4°C. Then, the supernatants were diluted with LC−MS grade water to a final concentration of 53% methanol. The samples were centrifuged at 15,000 × g and 4°C for 20 min. Finally, the supernatant was injected into the LC−MS/MS system for analysis. An equal volume of sample was taken from each experimental sample and mixed thoroughly to serve as the QC (Quality Control) sample.

### 2.3 UHPLC-MS/MS analysis and data analysis

UHPLC-MS/MS analysis were performed using a Vanquish UHPLC system (ThermoFisher, Germany) coupled with an Orbitrap Q Exactive™ HF mass spectrometer (Thermo Fisher, Germany) in Novogene Co., Ltd. (Beijing, China). Samples were injected onto a Hypersil Goldcolumn (100×2.1 mm, 1.9μm) using a 12-min linear gradient at a flow rate of 0.2 mL/min. The eluents for the positive polarity mode were eluent A (0.1% FA in Water) and eluent B (Methanol). The eluents for the negative polarity mode were eluent A (5 mM ammonium acetate, pH 9.0) and eluent B (Methanol).The solvent gradient was set as follows: 2% B, 1.5 min; 2-85% B, 3 min; 85-100% B, 10 min;100-2% B, 10.1 min;2% B, 12 min. Q Exactive™ HF mass spectrometer was L/min, S-lens RF level of 60, Aux gas heater temperature of 350°C.

The raw data files generated by UHPLC-MS/MS were processed using the Compound Discoverer 3.3 (CD3.3, ThermoFisher) to perform peak alignment, peak picking, and quantitation for each metabolite. The main parameters were set as follows: peak area was corrected with the first QC, actual mass tolerance, 5ppm; signal intensity tolerance, 30%; and minimum intensity, et al. After that, peak intensities were normalized to the total spectral intensity. The normalized data was used to predict the molecular formula based on additive ions, molecular ion peaks and fragment ions. And then peaks were matched with the mzCloud (https://www.mzcloud.org/), mzVault and MassList database to obtain the accurate qualitative and relative quantitative results. Statistical analyses were performed using the statistical software R (R version R-3.4.3), Python (Python 2.7.6 version) and CentOS (CentOS release 6.6), When data were not normally distributed, standardize according to the formula: sample raw quantitation value/(The sum of sample metabolite quantitation value/The sum of QC1 sample metabolite quantitation value)to obtain relative peak areas; And compounds whose CVs of relative peak areas in QC samples were greater than 30% were removed, and finally the metabolites’ identification and relative quantification results were obtained.

These metabolites were annotated using the KEGG database (https://www.genome.jp/kegg/pathway.html), HMDB database (https://hmdb.ca/metabolites) and LIPIDMaps database (http://www.lipidmaps.org/). Principal component analysis (PCA)and partial least-squares discriminant analysis (PLS-DA) were performed at metaX. We applied univariate analysis (t-test) to calculate the statistical significance (P-value). The metabolites with VIP > 1 and P-value < 0.05 and fold change (FC) ≥ 2 or FC ≤ 0.5 were considered to be differential metabolites.

### 2.4 RNA isolation

Fresh tissue samples of roots, leaves, and flowers (100 mg) were mixed with liquid nitrogen and ground into a fine powder. According to the manufacturer’s instructions, total RNA was extracted using an RNA isolation kit (Beijing Huayue Biotechnology Co., Ltd.). Then, 1.0% agarose gel electrophoresis, a NanoDrop ND-1000 (NanoDrop Technologies, DE, USA) and an Agilent Biological Analyzer 2100 system (Agilent Technologies) were used to detect and assess the total RNA concentration and quality.

### 2.5 Iso-seq sequencing and analysis

We mixed RNA from dandelion roots, leaves, and flowers to perform Iso-seq. The flowchart for constructing the Iso-seq library is shown in **Supplementary Figure S1**. The mRNA was reverse transcribed into full-length cDNA using a SMARTer PCR cDNA Synthesis Kit (Clontech, CA, USA). Then, cDNA amplification was performed using an Advantage 2 PCR Kit (Clontech, CA, USA). After amplification, each sample was screened for fragments using BluePippin (Sage Science) to enrich fragments with lengths exceeding 4 kb. The unscreened and size-selected cDNA fragments were prepared into SMRTbell template libraries using a SMRTbell Template Prep Kit (Pacific Biosciences, CA, USA). To prepare the sequencing library, the sequencing primers were annealed (using a component of the SMRTbell Template Prep Kit 1.0), and polymerase was bound to the primer annealing template. The polymerase-bound template was bound to MagBeads (P/N 100-125-900), and sequencing was performed on a PacBio RS II instrument at Novogene Bioinformatics Technology Co., Ltd. (Beijing, China). Circular consensus sequence (CCS) was used to identify the full-length nonchimeric (FLNC) and non-full-length nonchimeric (nFL) sequences based on whether they contained 5’-prime, 3’-prime, or poly-A sequences. After clustering with the hierarchical n*log(n) algorithm, cluster consensus sequences were obtained. Finally, the obtained full-length sequence was polished to obtain high-quality consensus sequences for subsequent analysis.

### 2.6 RNA-Seq sequencing and analysis

Three biological replicates of dandelion roots, leaves, and flowers were used for RNA-seq. The mRNA was transformed into cDNA using the TruSeq RNA Sample Prep Kit v2 (Illumina, San Diego, CA, USA), and sequencing was carried out on the Illumina NovaSeq 6000 platform (Illumina, San Diego CA, USA) (Novogene Company, www.novogene.com). The RNA-Seq data is used to correct the full-length transcriptome sequence of dandelion through the LoRDEC software. Differential expression analysis was performed using the DESeq2 R package (1.20.0). GO enrichment analysis of differentially expressed genes was implemented by the clusterProfiler R package, in which gene length bias was corrected. GO terms with corrected P-value < 0.05 were considered significantly enriched by differential expressed genes. We used clusterProfiler R package to test the statistical enrichment of differential expression genes in KEGG pathways.

### 2.7 Quantitative real-time PCR analysis

To validate the transcriptome data, sixteen unigenes (including 9 flavonoid biosynthesis-related genes and 7 chicoric acid biosynthesis-related genes) from the transcriptome database were selected for qRT−PCR. The specific primers for each gene were designed using Primer Premier 5.0 (**Supplementary Table S1**). Actin was used as an internal standard for mRNA expression in each sample. cDNA was synthesized using a Prime Script™ RT Reagent Kit (with gDNA Eraser) (TaKaRa, Japan). Then, qRT−PCR was performed using a Bio-Rad CFX96 real-time system (Bio-Rad, America) and TB Green^®^ *Premix Ex Taq* ^™^ (Tli RNaseH Plus) (Takara, RR420A) following the manufacturer’s instructions (TaKaRa Biotechnology). The dandelion actin gene was used as a reference gene for mRNA expression in each sample. The relative gene expression levels were calculated using the 2^− ΔΔCt^ method (Livak and Schmittgen, 2001). Statistical Product and Service Solutions (SPSS 11.5) was used to conduct the statistical analysis. Analysis of variance (ANOVA) was used to separate the means, and Duncan’s multiple range test was used to test for significant differences at P<0.05.

## 3 Results

### 3.1 Metabolomic profiling

**Figure 1:**
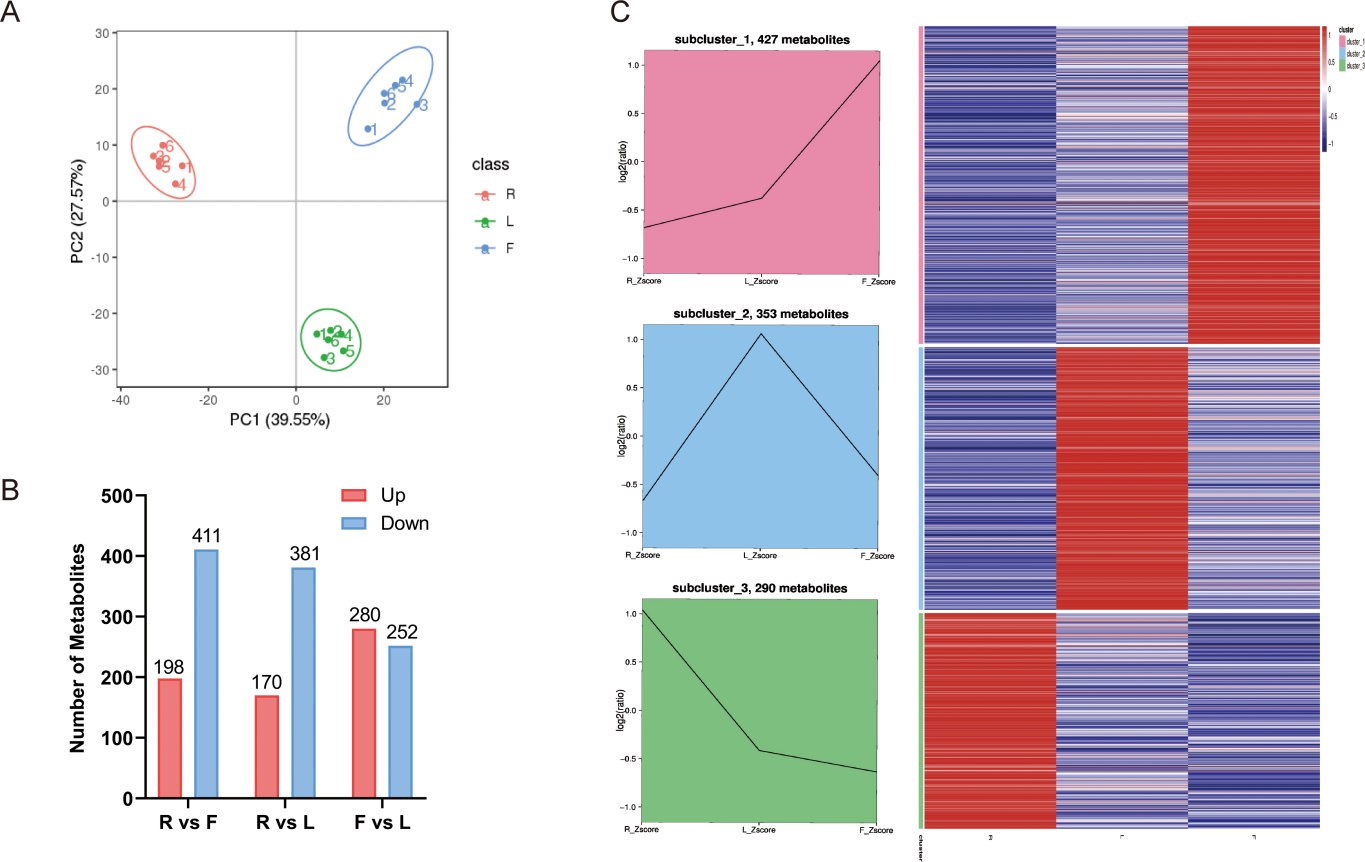
Differential metabolites analysis. **(A)** Principal component analysis (PCA) of LC–MS/MS results from different dandelion tissue samples; **(B)** Line chart plot and clustering heatmap of the K-means clustering analysis of the differential metabolites. **(C)** Statistics for the DAMs for the different comparisons (R vs. L, R vs. F, and F vs. L) in dandelion tissues.

We used an LC−MS/MS-based metabolic profiling method to analyze the metabolites in different dandelion tissues. The total ion chromatograms (TICs) of all the QC samples showed the same retention times and peak areas, and the high stability of the instrument ensured the reliability of the data (**Supplementary Figure S2**). Correlation analysis was conducted to evaluate biological replicates in each group, and all QC samples had good reproducibility (**Supplementary Figure S3**). A total of 1298 metabolites were identified in the R, L, and F samples. These metabolites included various classes, including 259 lipids and lipid-like molecules, 206 phenylpropanoids and polyketides, 132 organic acids and derivatives, 112 organoheterocyclic compounds, 102 organic oxygen compounds, 87 benzenoids, 56 nucleosides, nucleotides, and analogs, 17 lignans, neolignans and related compounds, 15 alkaloids and derivatives, 12 organic nitrogen compounds, 1 mixed metal/nonmetal compound, 1 homogeneous nonmetal compound, and 298 compounds that were not classified into any specific group (**Supplementary Table S2**). Eighty-eight flavonoids and twenty-five phenolic acids were identified (**Supplementary Table S3**). In this study, principal component analysis (PCA) was used to analyze the credibility of the identification results and investigate variations in the metabolite accumulation patterns of dandelion in different tissues (**Figure 1A**). PC1 and PC2 explained 39.55% and 27.57% of the total variance, respectively (**Figure 1A**). The results indicated significant differences in metabolites between different dandelion tissues. The heatmap clustering results showed significant differences in metabolite content among the F, R, and L samples (**Supplementary Figure S4**). These results indicated that the metabolome detection was highly reliable.

VIP ≥ 1 and FC ≥ 1.5 or ≤ 0.667 were used to screen for differentially accumulated metabolites (DAMs) in each comparison group. The DAMs of different dandelion tissues (R vs. L, R vs. F, F vs. L) were analyzed. A total of 1052 DAMs were obtained from the comparison group (**Supplementary Table S4**). The number of DAMs in each group is summarized in **Figure 1B**, and the DAMs for each comparison group are shown in **Supplementary Table S5**. There were 609, 551 and 532 DAMs in R vs. F, R vs. L, and F vs. L, respectively. In the R vs. F comparison, 198 DAMs were upregulated, and 411 were downregulated. A total of 170 and 381 DAMs were up- and downregulated, respectively, in the R and L comparison. A total of 280 DAMs were upregulated and 252 were downregulated in F compared to L. The results showed that the DAMs exhibited significant between R and L and between R and F, whereas the DAMs showed less differences between F and L. To analyze the trend of changes in all DAMs, K-means analysis was performed. K-means analysis divided the DAMs into three clusters (**Figure 1C**). Among them, 427 metabolites were significantly increased in F, mainly fatty acyls, carboxylic acids and derivatives, prenol lipids, organooxygen compounds, steroids and steroid derivatives, and flavonoids. A total of 353 metabolites, including fatty acyls, cinnamic acids and derivatives, benzene and substituted derivatives, prenol lipids, organooxygen compounds, phenols, steroids and steroid derivatives, isoflavonoids, coumarins and derivatives, and flavonoids, were significantly increased in L. A total of 290 metabolites were significantly increased in R, mainly indoles and derivatives, carboxylic acids and derivatives, benzene and substituted derivatives, prenol lipids, organooxygen compounds and flavonoids (**Supplementary Table S5**).

### 3.2 Full-length transcriptome analysis of dandelion

**Figure 2:**
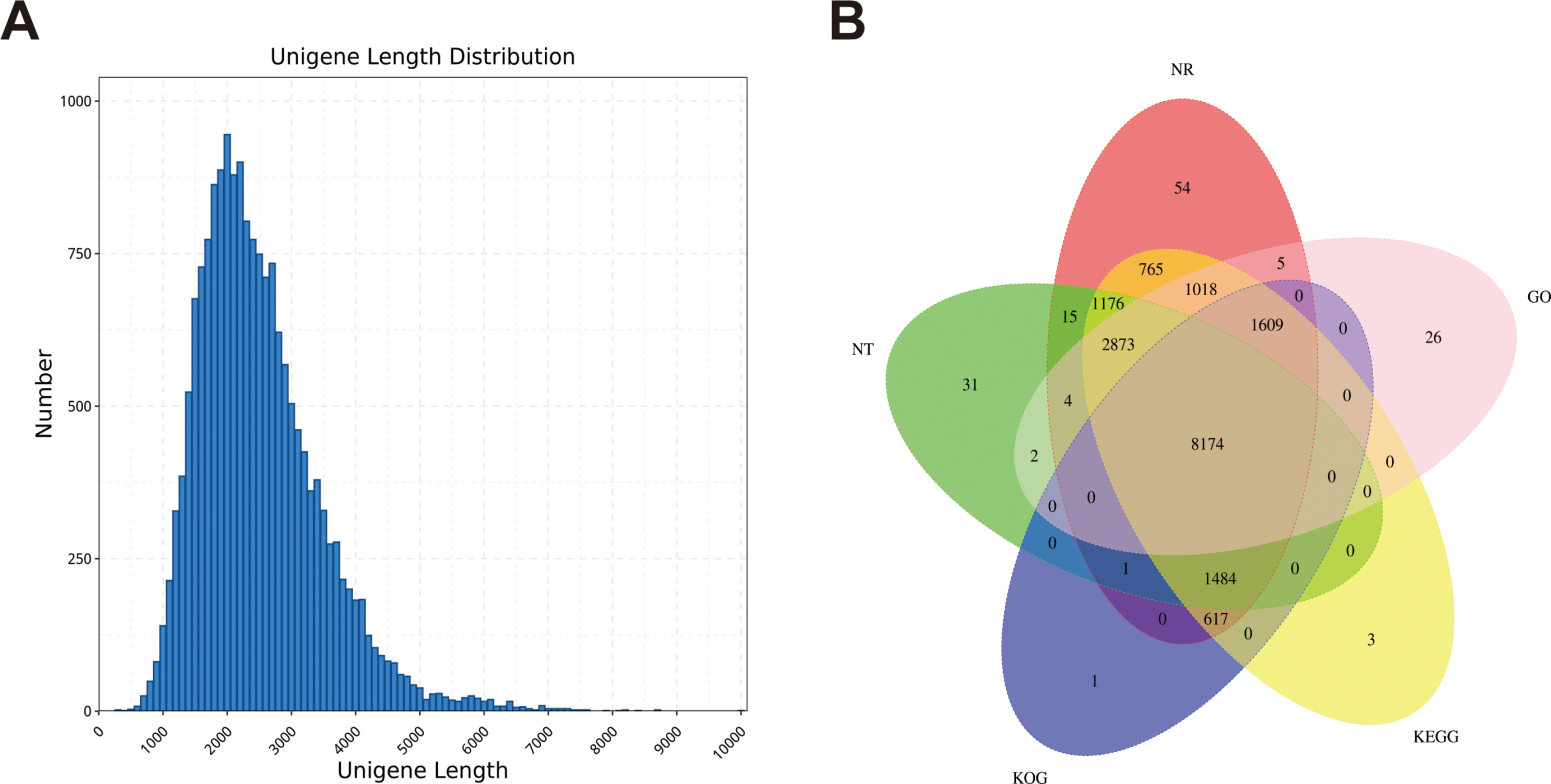
Construction of the full-length transcriptome of dandelion tissues. **(A)** The length distribution of genes. **(B)** Annotation of genes to the NR, Pfam, SwissProt, KOG, and KEGG databases.

We obtained 64.96 Gb of data containing 23834203 subunits for the full-length transcriptome using the PacBio Sequel sequencing platform (**Supplementary Table S7**). Among the 18346 genes, 18032 were annotated with at least one hit in the NR, SwissProt, KEGG, KOG, GO, NT and Pfam databases (**Supplementary Table S8**). Five public protein databases shared 8174 matched genes (**Figure 2B**). Among them, in the Nr database, the five species with the best BLAST hits for dandelion genes were *Cynara cardunculus*, *Vitis vinifera*, *Daucus carota*, *Sesamum indicum* and *Coffea canephora* (**Supplementary Figure S5**). More than half of the *Cynara cardunculus* genes were associated with dandelion, indicating a high degree of homology between *Cynara cardunculus* and dandelion, as they are both members of the Asteraceae family. According to the GO database, 57715 genes were classified into biological processes, cellular components, and molecular function. For biological processes, the first three subgroups were single organic process (3842), cellular process (6295), and metabolic process (6656). For cellular components, the first three subgroups were organelle (1769), cell part (2519), and cell (2519). For molecular function, the first three subgroups were binding (9083), transporter activity (769), and catalytic activity (6854) (**Supplementary Figure S6**). According to the KOG database, 13515 genes were classified into 25 functional categories, with the largest and smallest categories being “general function prediction only” (1584 genes) and “cell motility” (42 genes), respectively (**Supplementary Figure S7**). According to the KEGG database, 15127 genes were annotated in six branches (cellular processes, environmental information processing, genetic information processing, human disappearances, metabolism, and organizational systems), with the highest proportion of genes included in “signal translation” of the environmental information processing branch (**Supplementary Figure S8**). In this study, a total of 18594 CDSs were predicted, with a length range of 400-2600 nt being the most common (**Figure 2A**; **Supplementary Figure S9**). We identified 986 TFs distributed in 28 families (**Supplementary Figure S10**). These data provide a rich and accurate transcript pool for further research on a series of biological issues related to dandelion.

### 3.3 Transcriptome analysis of dandelion

**Figure 3:**
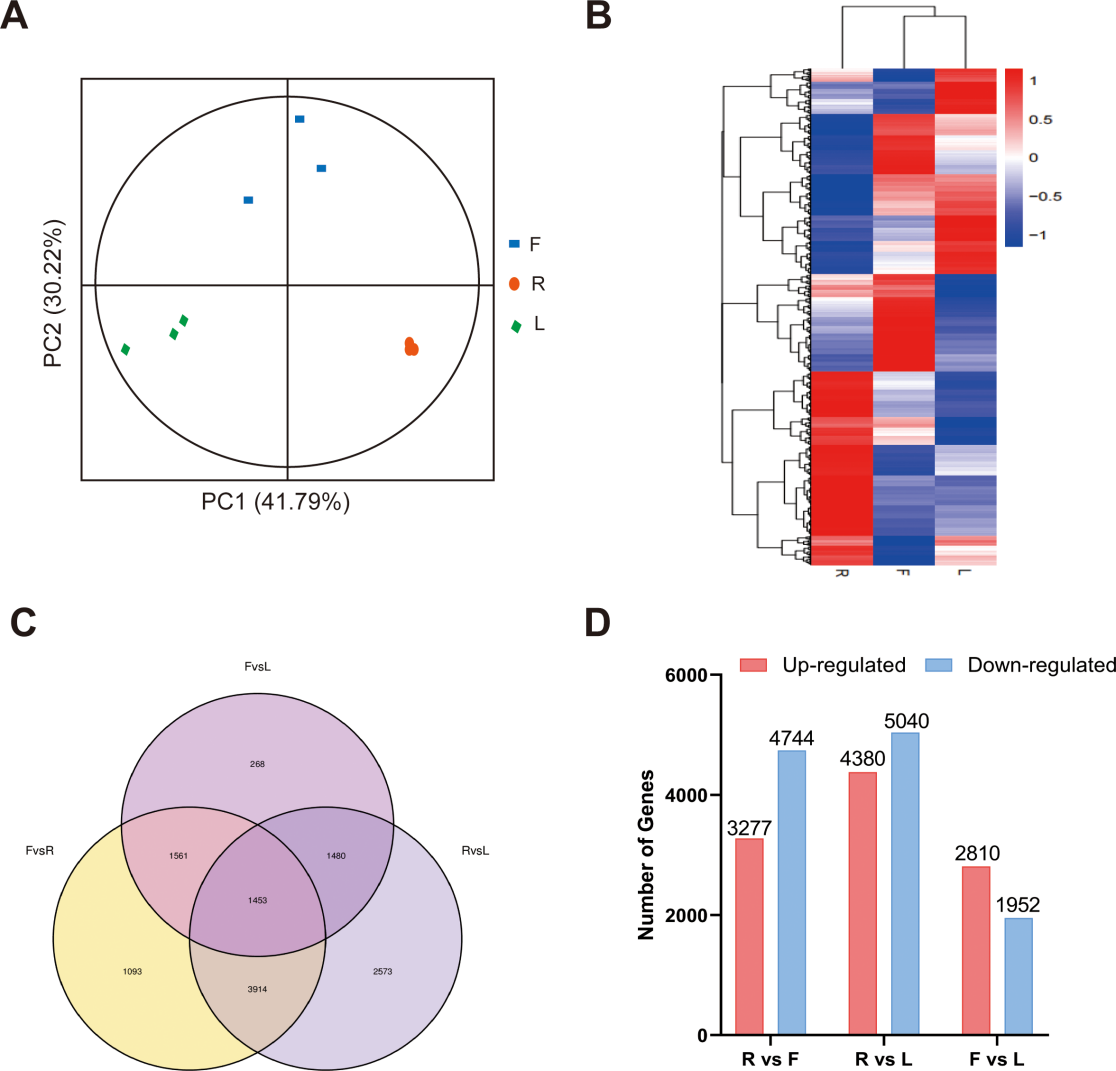
RNA-seq data expression profiles of dandelion tissues. **(A)** PCA of transcriptome data from the samples of three dandelion tissues. **(B)** Heatmap based on the hierarchical clustering analysis. **(C)** Venn diagram of the DEGs for the different comparisons. **(D)** The number of up- and downregulated DEGs in the different comparisons.

For transcriptome analysis, we used fragments per kilobase per million fragments (FPKM) values to standardize the expression levels. We detected the expression of 18164 genes in at least one sample, and the FPKM value of each sample is shown in **Supplementary Table S9**. According to the PCA and clustering heatmaps, the clustering of different tissues in the transcriptome was consistent with that in the metabolome (**Figures 3A, B**). These results indicated that the accumulation of metabolites and gene expression were highly specific in different dandelion tissues. We conducted a comparative analysis of the DEGs in the roots, flowers, and leaves (**Figures 3C, D**). The Venn diagram indicated that most of the specifically expressed genes were found in the comparison of R and L (2573), while the F and L comparison had the fewest differentially expressed genes (268), indicating a significant biological difference between R and L. A total of 1453 genes were differentially expressed in all comparison groups, indicating that these genes play a crucial regulatory role in the growth and development of dandelion (**Figure 3C**). A total of 8021, 9420, and 4762 DEGs were identified in the R vs. F, R vs. L, and F vs. L comparisons, respectively (**Figure 3D**). The DEGs identified in the three comparisons were selected as candidate genes related to polyphenol biosynthesis in dandelion, and further functional analysis was conducted.

**Figure 4:**
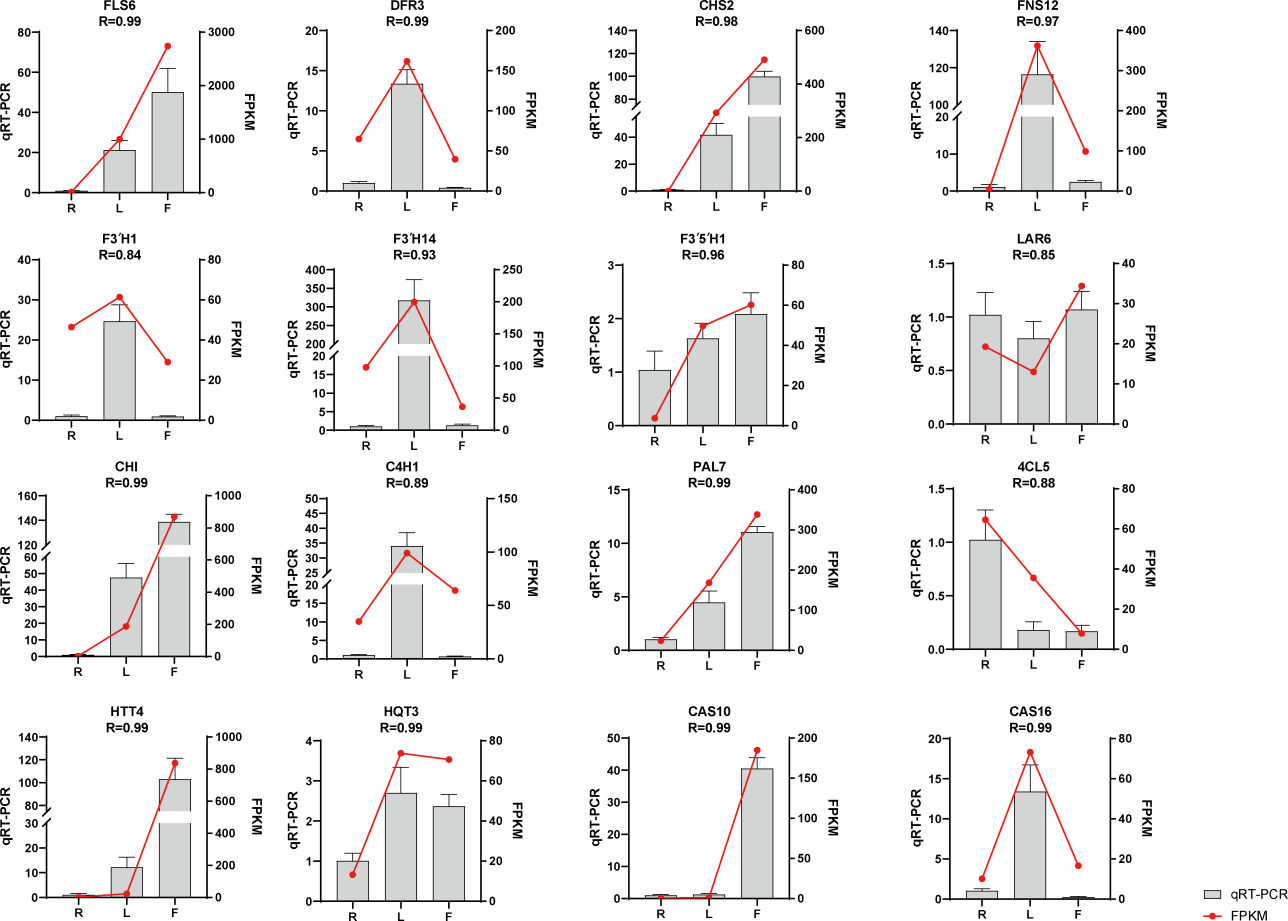
Expression of flavonoid- and chicoric acid biosynthesis-related genes quantified by RNA-seq and qRT–PCR analyses. The y-axis represents the FPKM values of the genes from the RNA-seq data and the relative gene expression levels analyzed by qRT–PCR. The error bars indicate the standard error for three independent replicates.

We selected sixteen DEGs for qRT−PCR analysis to verify the accuracy of the transcriptome data. The results showed that the qRT−PCR and transcriptome data were basically consistent (**Figure 4**), with R > 0.84, indicating that the transcriptome sequencing data were reliable.

### 3.4 Analyses of the DEGs involved in flavonoid biosynthesis pathways in different tissues

**Figure 5:**
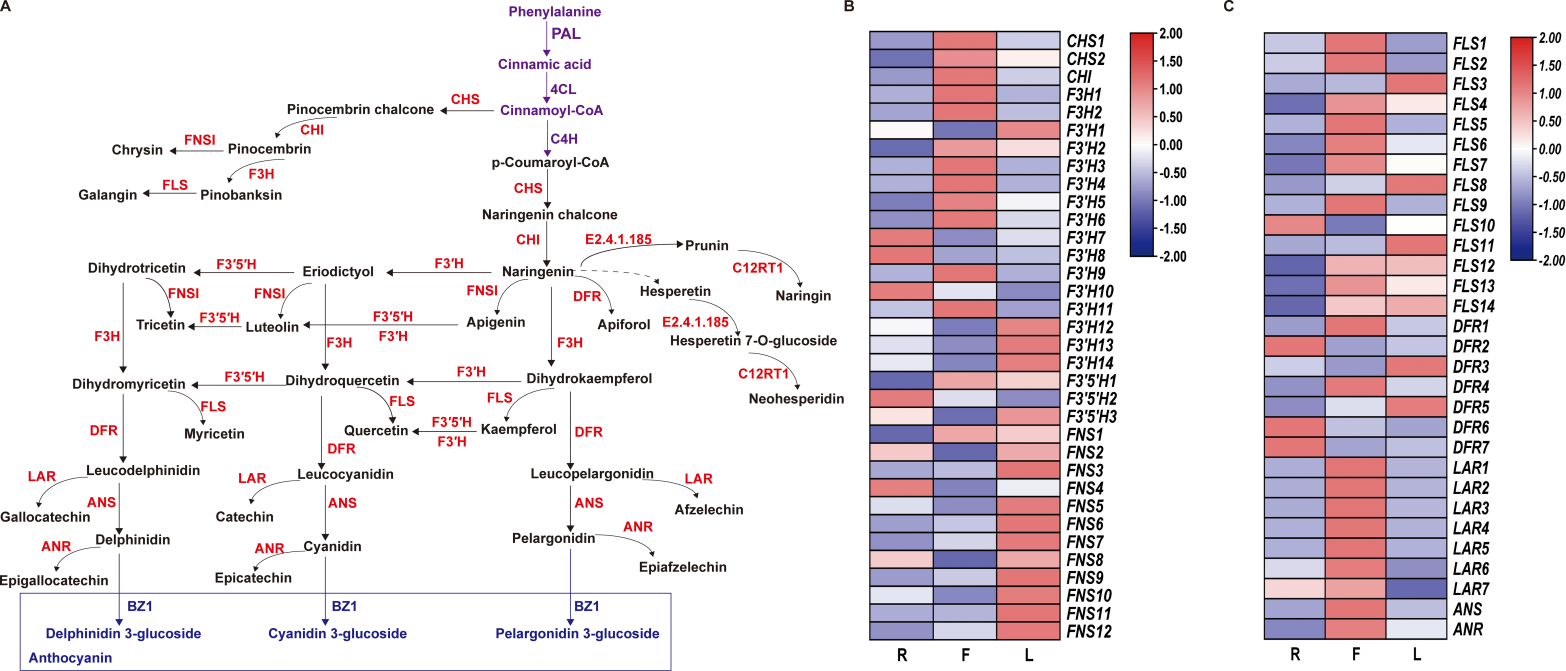
Biosynthesis of flavonoids in dandelion. **(A)** Simplified diagram showing the flavonoid biosynthetic pathways. **(B, C)** Heatmaps of the relative expression of genes encoding enzymes involved in flavonoid synthesis in different tissues as created using TBtools with ‘log scale’ and ‘row scale’. Chalcone synthase (CHS), chalcone isomerase (CHI), flavanone 3-hydroxylase (F3H), flavonoid 3′-hydroxylase (F3′H), flavonoid 3′5′-hydroxylase (F3′5′H), flavonol synthase (FLS), flavone synthase (FNS), dihydroflavonol 4-reductase (DFR), and anthocyanidin synthase (ANS) were identified.

We constructed a dandelion flavonoid biosynthesis pathway by referring to the flavonoid biosynthesis pathways in the KEGG database and detected genes and metabolites (**Figure 5A**). A total of 64 genes were identified from 11 gene families involved in the flavonoid biosynthesis pathway (**Figures 5B, C**), including 2 CHS genes, 1 CHI gene, 2 F3H gene, 14 F3′H gene, 3 F3′5′H gene, 12 FNS gene, 14 FLS gene, 7 DFR gene, 7 LAR gene, 1 ANS gene, and 1 ANR gene. As shown in **Figures 5B, C**, 34 genes were specifically expressed in F, which was more genes than in the other two tissues. In this pathway, metabolites (kaempferol, 5-O-caffeoylshikimic acid, apigenin, taxifolin, chrysin, luteolin, naringenin chalcone, galangin, rhoifolin, rutin and syringetin) were significantly accumulated in F, which may be related to the high gene expression in F (**Figures 5B, C**; **Supplementary Table S10**). This finding indicates that F is the main tissue responsible for flavonoid synthesis and accumulation. However, 27 DEGs were downregulated in F, which may be due to the complex regulatory process of secondary metabolites. Compared to those in the other two tissues, the expression level of F3’5’H in F was lower, but the high expression of F3’H and F3H in F led to an increase in the content of luteolin, apigenin and kaempferol (**Figure 5**). This indicates that F3’H and F3H are the main hydroxylases in dandelion. The accumulation of flavonoid metabolites was mainly consistent with the greater upregulation of genes in F (**Figure 5**). These results indicate that there is a complex regulatory network between flavonoid accumulation and gene expression levels; further research is needed to validate the key genes involved in flavonoid biosynthesis in dandelion.

### 3.5 Analyses of the DEGs involved in chicoric acid biosynthesis pathways in different tissues

**Figure 6:**
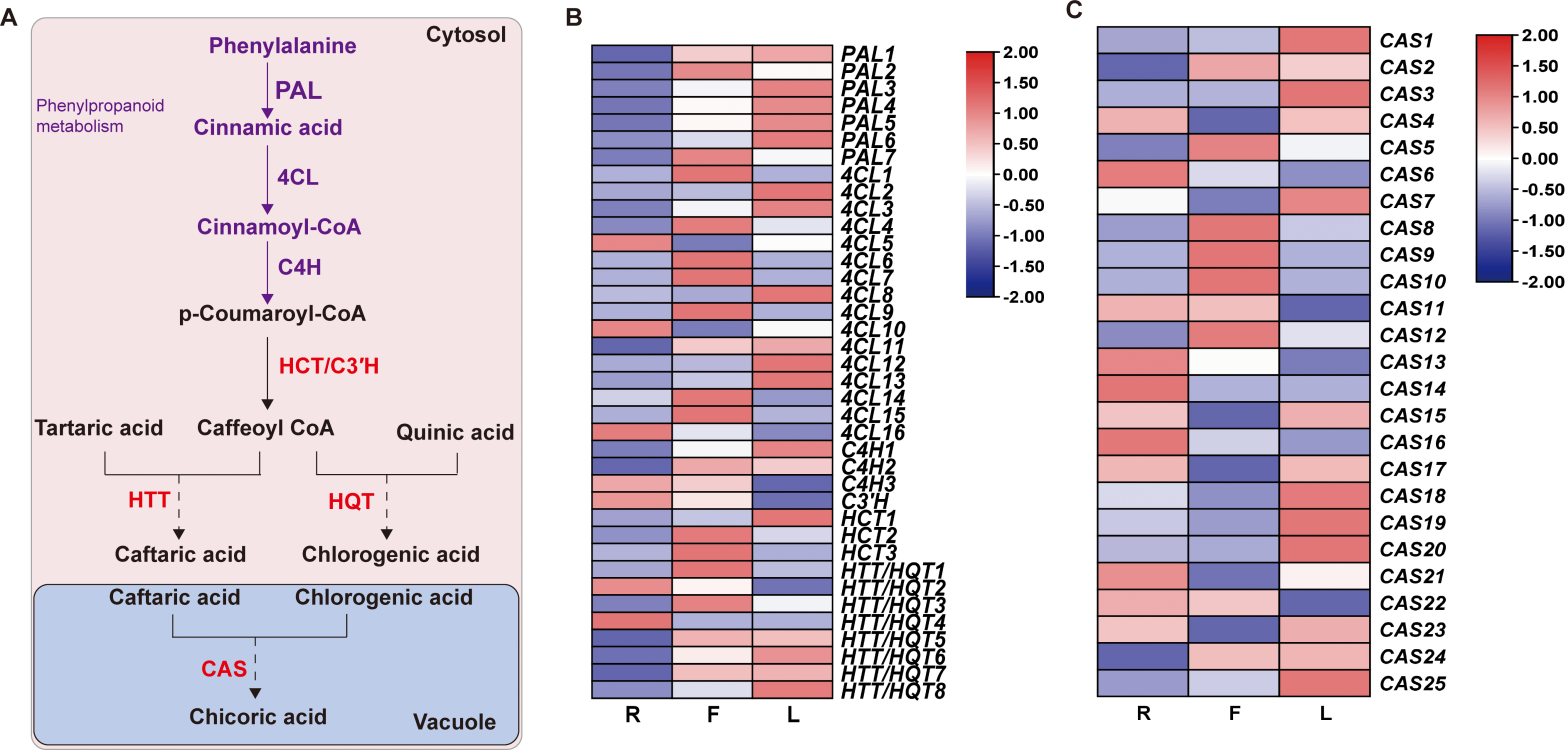
Biosynthesis of chicoric acid in dandelion. **(A)** Chicoric acid metabolic pathway. **(B, C)** Heatmaps of the relative expression of genes related to chicoric acid synthesis in different tissues as created using TBtools with ‘log scale’ and ‘row scale’. Phenylalanine ammonia-lyase (PAL), cinnamate 4-hydroxylase (C4H), 4-coumarate CoA ligase (4CL), hydroxycinnamoyl-coenzyme A: quinate/shikimate hydroxycinnamoyl transferase (HCT), 5-O-(4-coumaroyl)-D-quinate 3’-monooxygenase (C3’H), hydroxycinnamoyl-CoA: quinate hydroxycinnamoyl transferase (HQT), hydroxycinnamoyl-CoA: tartaric acid hydroxycinnamoyl transferase (HTT) and chicoric acid synthase (CAS) were identified.

To further explore the mechanism of dandelion chicoric acid accumulation, we analyzed the expression patterns of genes involved in the biosynthesis pathways of phenylpropane and chicoric acid. The phenylpropanoid pathway provides precursors for the biosynthesis of chicoric acid. PAL, 4CL, and C4H catalyze the production of sufficient precursors, followed by different downstream reactions catalyzed by HCT/HQT/HTT from the BAHD family and CAT from the SCPL family, leading to the biosynthesis of chicoric acid [**Figure 6** (Fu et al., 2021; Liu et al., 2023a)]. Based on amino acid sequence screening, we identified 63 genes encoding chicoric acid biosynthesis enzymes, including 7 PAL, 16 4CL, 3 C4H, 3 HCT, C3’H, 8 HQT/5 HTT, and 25 CAS (**Supplementary Table S10**). The high expression of PAL and C4H in L led to the greater content of the precursor substance p-coumaric acid in L (**Figure 6B**; **Supplementary Table S6**). In addition, the specific expression of HQT in F may be the reason for the high proportion of chlorogenic acid in F (**Figure 6B**; **Supplementary Table S6**). Compared to those in the other two tissues, 9 CAS genes were highly expressed in L, but the chicoric acid content in L was the lowest, indicating the need for further research and validation of the key genes responsible for the increase in chicoric acid content caused by these CASs.

## 4 Discussion

Dandelion is distributed worldwide and has a long medicinal history (Fan et al., 2023). It is thus necessary to study the dandelion varieties planted in China to guide the cultivation, development, and utilization of dandelion plants in the region. Polyphenolic compounds are important pharmacological compounds in dandelion, with varying levels in different tissues (González-Castejón et al., 2012), but their potential molecular mechanisms remain unclear. To reveal these differences, we selected the roots (R), leaves (L), and flowers (F) of stable and hereditary dandelion plants as our research materials. We combined full-length transcriptome, second-generation transcriptome, and nontargeted metabolome analyses to investigate the differential genes and metabolites of polyphenolic compounds in different dandelion tissues. In this study, 88 flavonoids and 25 phenolic metabolites were identified in dandelion. Subsequently, functional analysis was conducted to identify candidate genes for differences in polyphenolic compound content. We identified 64 candidate genes involved in flavonoid biosynthesis and 63 candidate genes involved in chicoric acid biosynthesis.

Currently, many flavonoids and phenolic acids have been identified in dandelion (Martinez et al., 2015); however, there are still many unidentified metabolites. Plant metabolomics has been widely used to detect and evaluate changes in metabolites in different tissues, species, or developmental stages (Li et al., 2018; Zou et al., 2021). In this study, nontargeted metabolomics analysis identified 1298 metabolites, including 88 flavonoids and 25 phenolic acids, in different dandelion tissues. In addition, 70 differentially accumulated flavonoids (including luteolin, kaempferol, rutin, quercetin, diosmetin, and apigenin) and 22 differentially accumulated phenolic acids (including chicoric acid, chlorogenic acid, ferulic acid, caffeic acid, gallic acid, and salicylic acid) were identified (**Supplementary Table S4**). Previous studies have reported that there are certain differences in the biological activity of different dandelion tissues (González-Castejón et al., 2012). The antioxidant content in different dandelion tissues is significantly different (Chon et al., 2012; García-Carrasco et al., 2015). Most of the polyphenolic compounds in F were more abundant than those in the other two tissues. The differences in polyphenolic compounds content may be related to the differences in upstream metabolites and enzymes in different parts of the plant. The index component of dandelion is chicoric acid, and the content of chicoric acid in F is significantly higher than that of the other tissues, providing a theoretical basis for the development of products using dandelion as a raw material. Research has shown that the phenolic components of dandelion flowers have stronger antioxidant properties than those of leaves (Jędrejek et al., 2017). This may be due to the higher phenolic content in flowers, which leads to stronger antioxidant properties.

Second-generation sequencing technology has been widely applied in research fields to obtain information on plant transcripts and determine quantitative gene expression. In the past few years, some researchers have used second-generation sequencing techniques to obtain information on dandelion transcripts to study the effects of grazing on dandelion gene expression (Wang et al., 2022), the regulation of abscisic acid (ABA) treatment on chicoric acid biosynthesis (Liu et al., 2023a), and the impact of jasmonic acid (JA) on dandelion signaling pathways (Verhoeven et al., 2018). However, due to the short sequencing fragments generated by second-generation sequencing technology, there are obstacles in transcriptome assembly and annotation (Bernard et al., 2014). Third-generation sequencing technology can generate longer reads than second-generation sequencing, and when combined with second-generation sequencing technology, more accurate and rich dandelion transcriptome data can be obtained. For example, the biosynthesis of olive polyphenols and their related gene expression patterns were studied by combining second-generation and third-generation transcriptome sequencing techniques (Guodong et al., 2019). Although recent studies on the biosynthesis of polyphenols in dandelion have been reported (Liu et al., 2018, 2019; Minling et al., 2022), the genes involved in the biosynthesis of polyphenols in different dandelion tissues remain unclear. In this study, Iso-seq and RNA-seq were performed on three different dandelion tissue samples. We obtained abundant transcriptome data, with a greater number of identified annotated unigenes than previously reported for transcriptome sequencing of dandelion roots, leaves, and flowers (Li et al., 2021). Differential expression analysis revealed that the number of DEGs in the R vs. L and F vs. R comparisons was greater than 8000. The high-quality reference transcriptome data in our research provide a solid foundation for subsequent research on dandelion.

The polyphenols in dandelion are mainly divided into phenolic acids and flavonoids. Polyphenols have many functions in plants, including protecting them from biotic and abiotic stresses such as wounding stress, drought and pathogen invasion (Deng and Lu, 2017; Khakdan et al., 2018; Yoshikawa et al., 2018; Guo et al., 2019). A total of 64 candidate genes involved in flavonoid biosynthesis and 63 candidate genes involved in chicoric acid biosynthesis were identified. Differential expression analysis revealed that the expression of 34 flavonoid biosynthesis-related genes and 29 chicoric acid biosynthesis-related genes was greater in F than that in the other two tissues. Multiple genes regulate the biosynthetic pathways of flavonoids and chicoric acids.

In tree peony, the high expression of PsF3H, PsF3’H, and PsFLS also promotes the synthesis of flavonoids (Luo et al., 2021). In addition, in pea plants, the high expression of the CHI, CHS, C4H, F3’5’H, F3H and F3’H genes leads to an increase in flavonoid content, and these enzymes are crucial for the synthesis of flavonoid compounds (Fu et al., 2023). Dandelion flowers presented the highest content of flavonoid compounds (**Figure 1**), and the antioxidant activity of dandelion flowers may be attributed to the presence of luteolin (Hu and Kitts, 2003). Li et al. (2021) reported that flowers accumulate significantly more luteolin than leaves or roots. RNA-seq analysis revealed 16 DEGs associated with luteolin biosynthesis, and F3’H might play a crucial role in luteolin biosynthesis. Therefore, the biosynthesis of luteolin is worthy of further study. This study identified 64 genes involved in flavonoid biosynthesis (including caffeoylshikimic acid, apigenin, taxifolin, chrysin, luteolin, naringenin chalcone, galangin, rhoifolin, rutin and syringetin). In F, the accumulation of flavonoids was increased, which may be attributed to the high expression of CHI, CHS, F3H and F3’H genes in the tissue. This result indicated that the high expression of CHS, CHI, F3H, F3’H, and FLS is crucial for the accumulation of flavonoids (flavones, flavonoids, and flavonols) in dandelion. We obtained more dandelion flavonoid pathway transcripts than previous studies (Li et al., 2021), indicating that the combination of second-generation and third-generation sequencing can increase data acquisition.

Previous studies have shown that HQT is involved in catalyzing the biosynthesis of chicoric acid (Liu et al., 2021). However, there is a lack of detailed information on the biosynthesis of chicoric acid in dandelion. In this study, 63 synthase genes involved in chicoric acid biosynthesis were identified (**Figure 6**). Based on amino acid sequence screening (MT936803.1, HTT; QRI59127.1, HQT; **Supplementary Table S11**), we identified 8 HQT/HTT. As the HQT/HTT domain belongs to the BAHD family and cannot be distinguished, functional validation is required for further *in vitro* research (Fu et al., 2021). Full-length transcriptome studies have shown that dandelion has high homology with *Cynara cardunculus* and *Vitis vinifera*. This study screened 31 CAS synthase genes (SCPL family), and further experiments are needed to confirm the functions of these structural genes *in vivo* and *in vitro*.

## 5 Conclusion

In summary, this study revealed correlations between polyphenol metabolites and genes in dandelion roots, leaves, and flowers. We identified 1298 metabolites from three tissues of dandelion plants, including 88 flavonoids and 25 phenolic acids. By combining the second-generation and third-generation transcriptomes, the genes of two main polyphenol biosynthesis pathways (flavonoids and chicoric acid) were analyzed. We identified 64 candidate genes involved in flavonoid biosynthesis and 63 candidate genes involved in chicoric acid biosynthesis. These results can help us better understand the biosynthetic pathways of polyphenols in dandelion, providing valuable resources for future molecular breeding and metabolic engineering research in dandelion.

## Data Availability

The full-length transcriptome and second-generation transcriptome data for this study are available from the NCBI Sequence Read Archive (SRA) database with the following accession number: PRJNA1117579 and PRJNA1117572.

## Supplementary material

Supplementary Table 1 Primers used in this study.

Supplementary Table 4 Names and Z scores of 1052 DAMs.

Supplementary Table 6 K-means analysis of DAMs.

Supplementary Table 8 Number of genes annotated in the NT, NR, Swiss-Prot, KEGG, KOG, Pfam, and GO databases.

Supplementary Table 9 The FPKM value of each sample.

Supplementary Table 10 Quantitative values (peak area values) of mass spectrometry detection of flavonoids in different tissues.

Supplementary Table 11 Chicoric acid biosynthetic genes and their expression levels in the transcriptome of dandelion.
