# Supplementary material for: Integrative analysis of the metabolome and transcriptome reveals the mechanism of polyphenol biosynthesis in Taraxacum mongolicum
Source: Front Plant Sci. 2024 Aug 16;15:1418585. doi: 10.3389/fpls.2024.1418585 (PMC11361933; doi:10.3389/fpls.2024.1418585)
Supplement: Supplementary file 2 [file DataSheet1.docx]

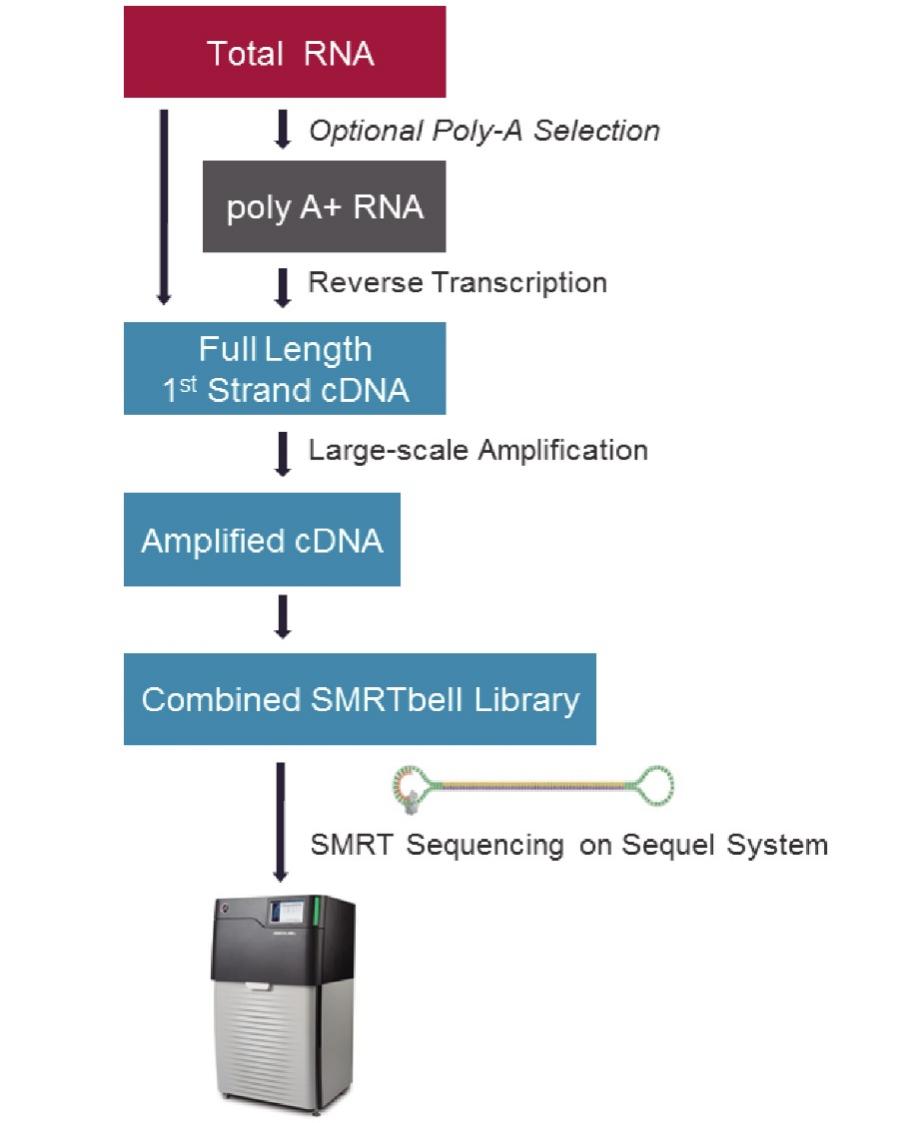


Supplementary Fig. S1 The flowchart for constructing the Iso-seq library.


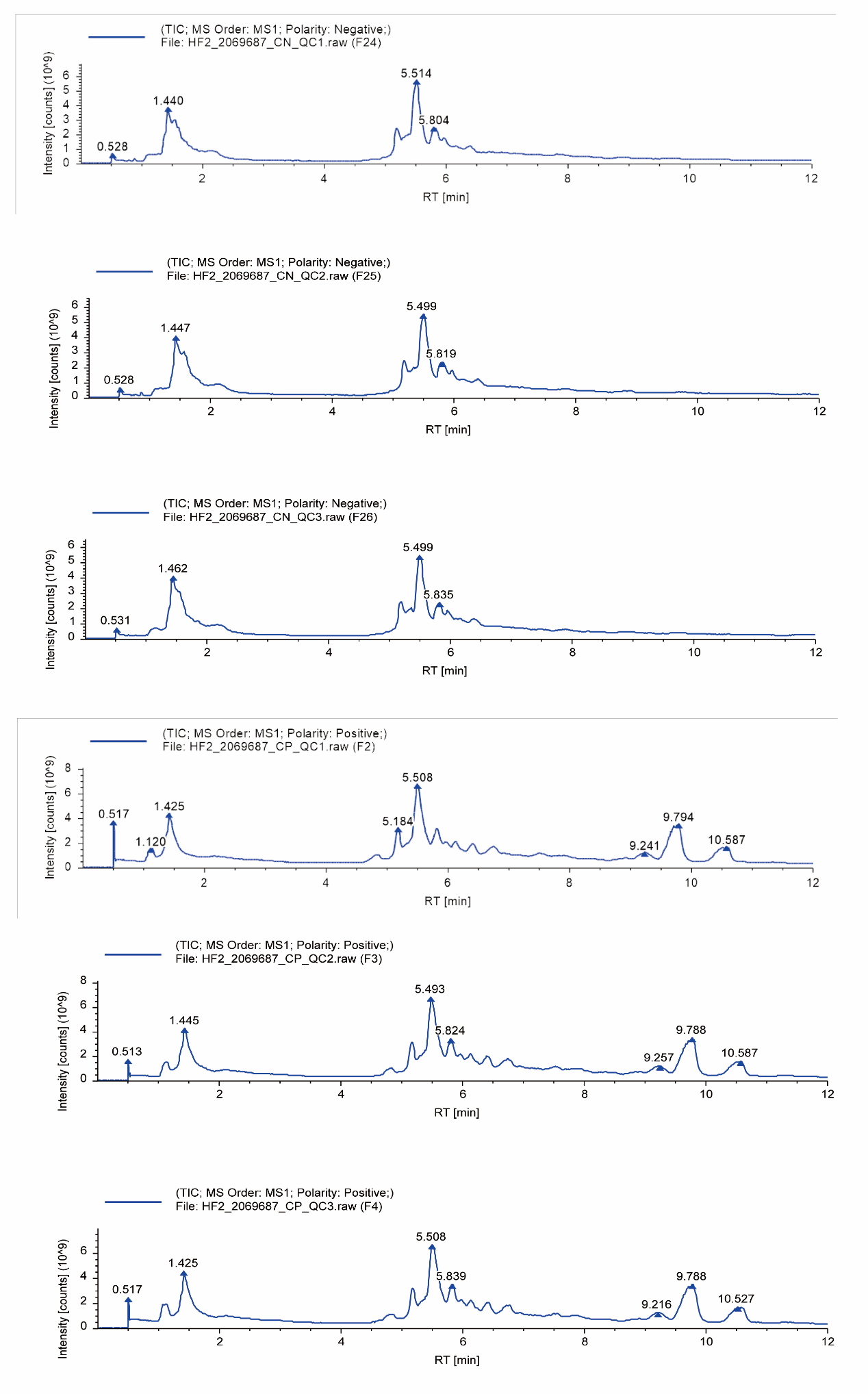


Supplementary Fig. S2 Total Ion Current (TIC) of QC samples in negative and positive ion mode.


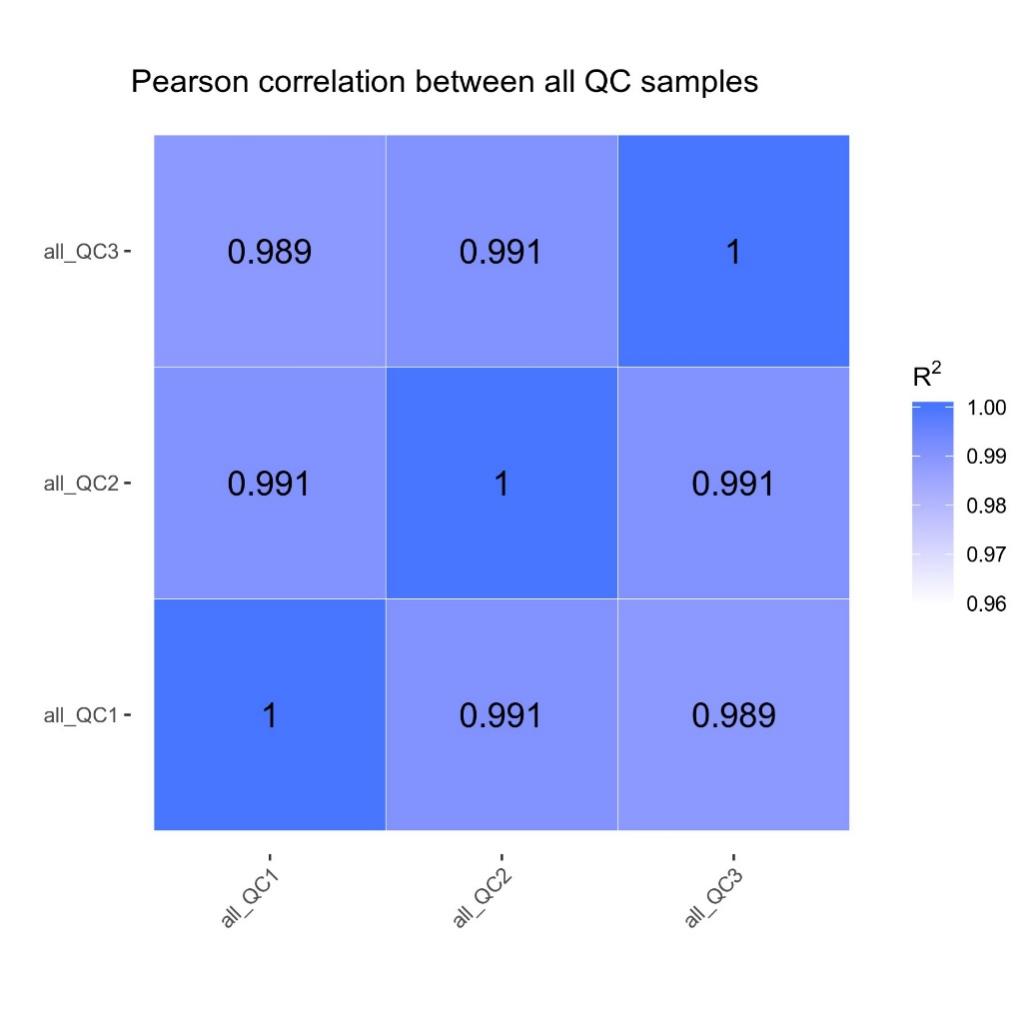


Supplementary Fig. S3 The correlation analysis using Pearson’s Correlation Coefficient (PCC).


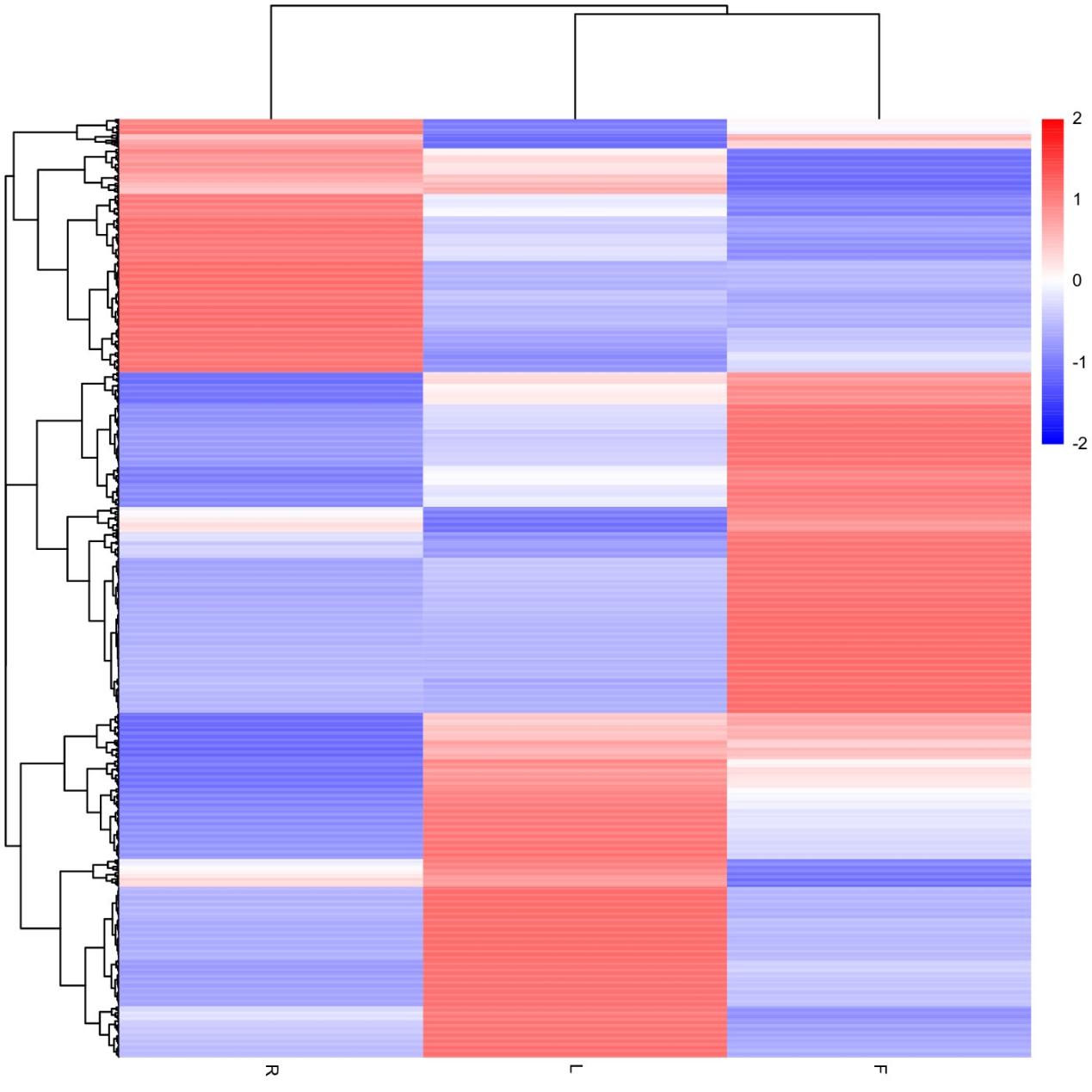


Supplementary Fig. S4 Heatmap based on hierarchical clustering analysis.


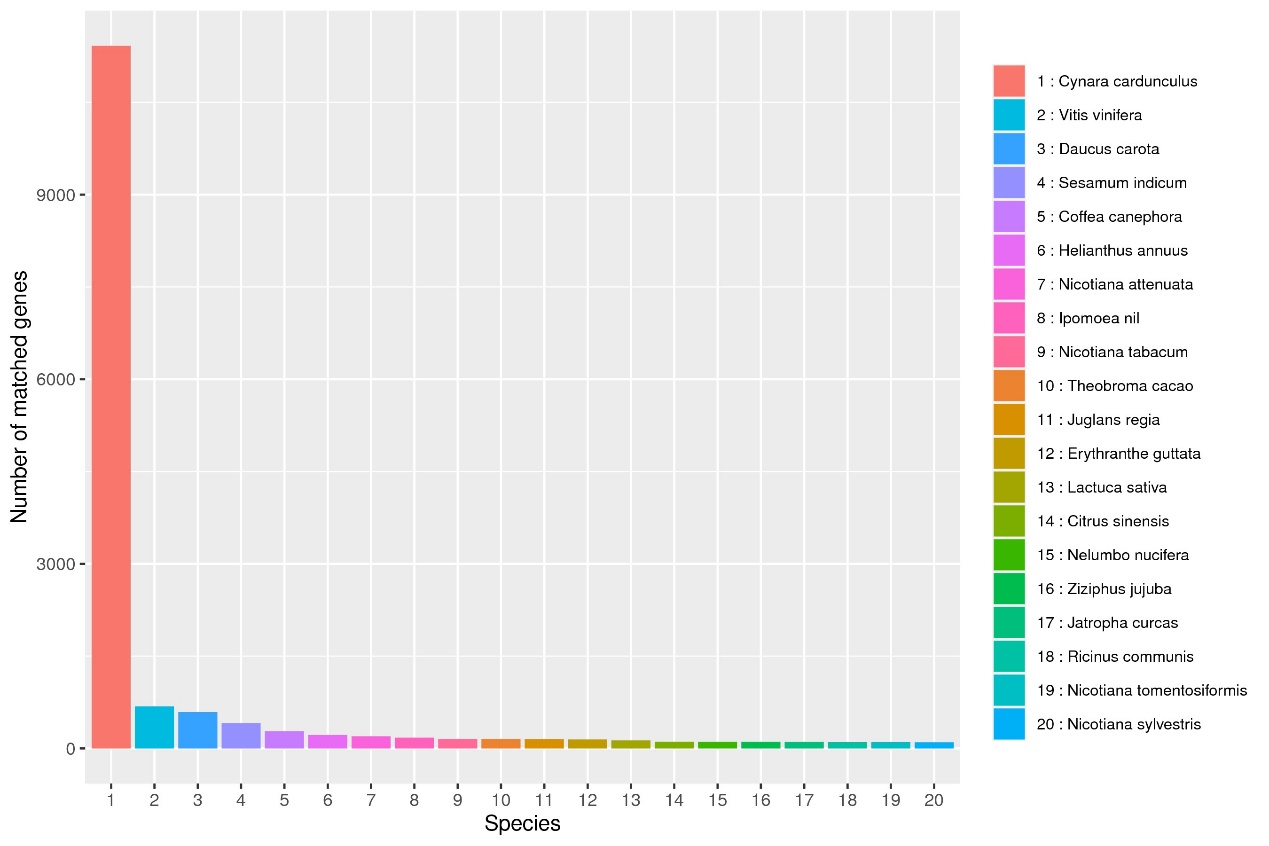


Supplementary Fig. S5 The matched species of annotation of the genes of dandelion to Nr database.


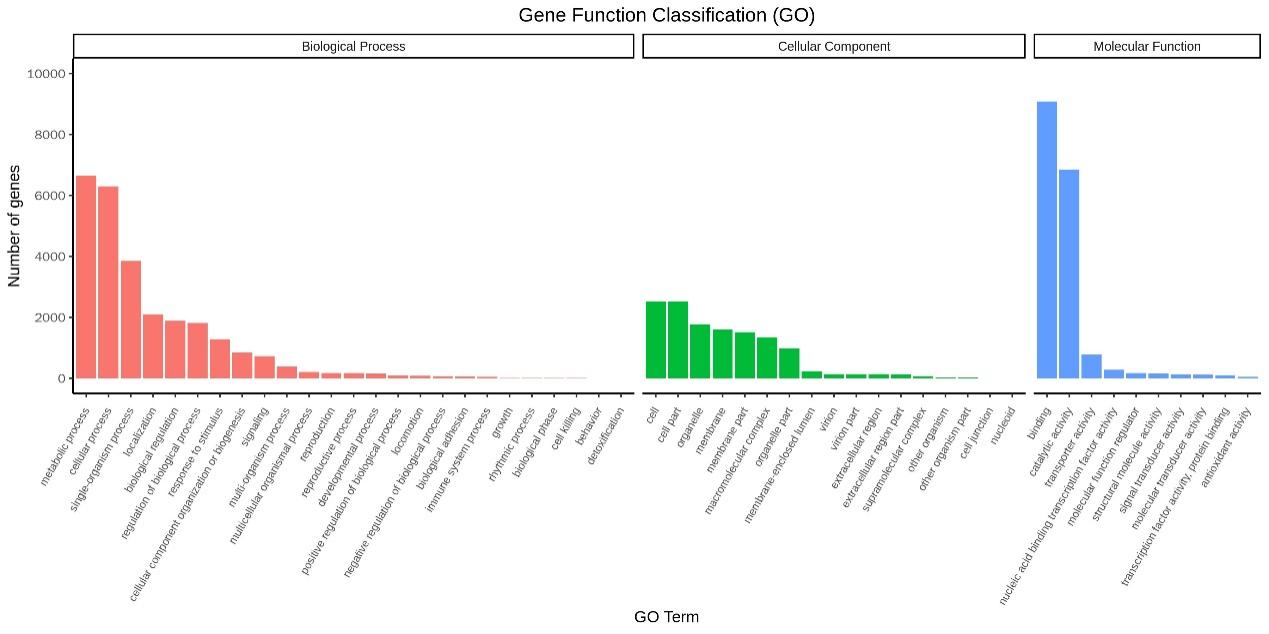


Supplementary Fig. S6 GO annotation of the genes of dandelion.


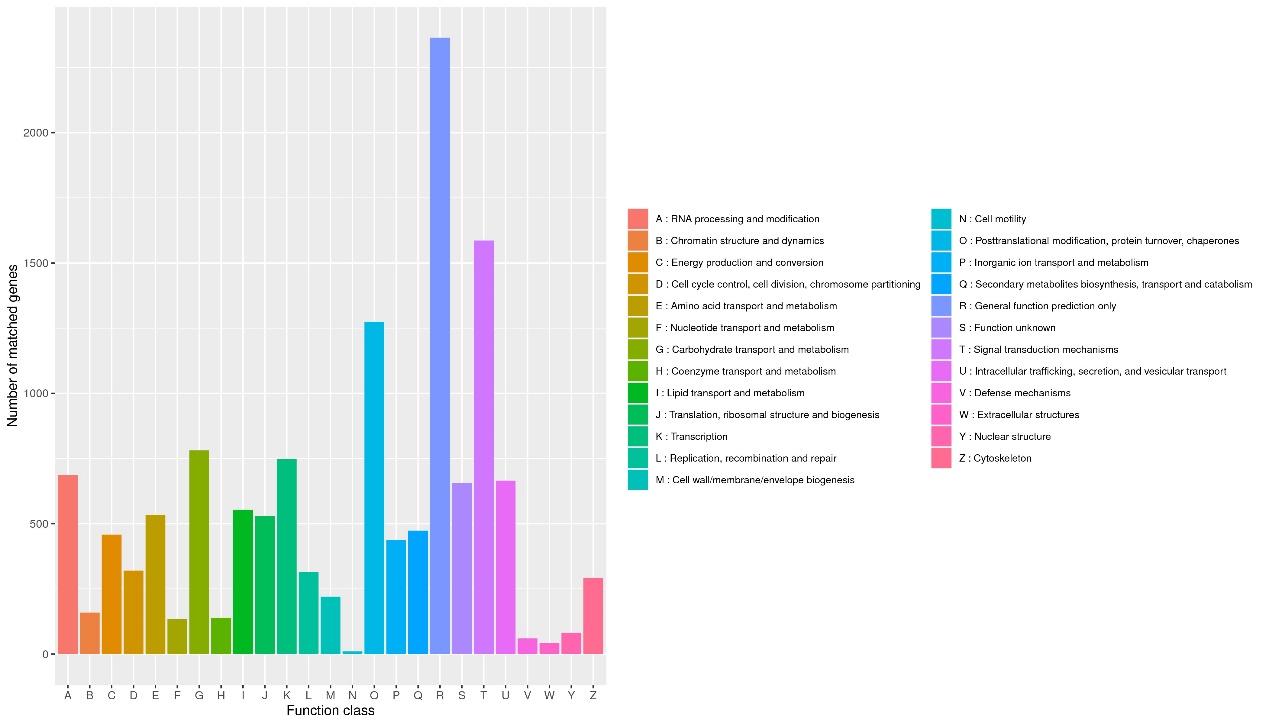


Supplementary Fig. S7 KOG annotation of the genes of dandelion.


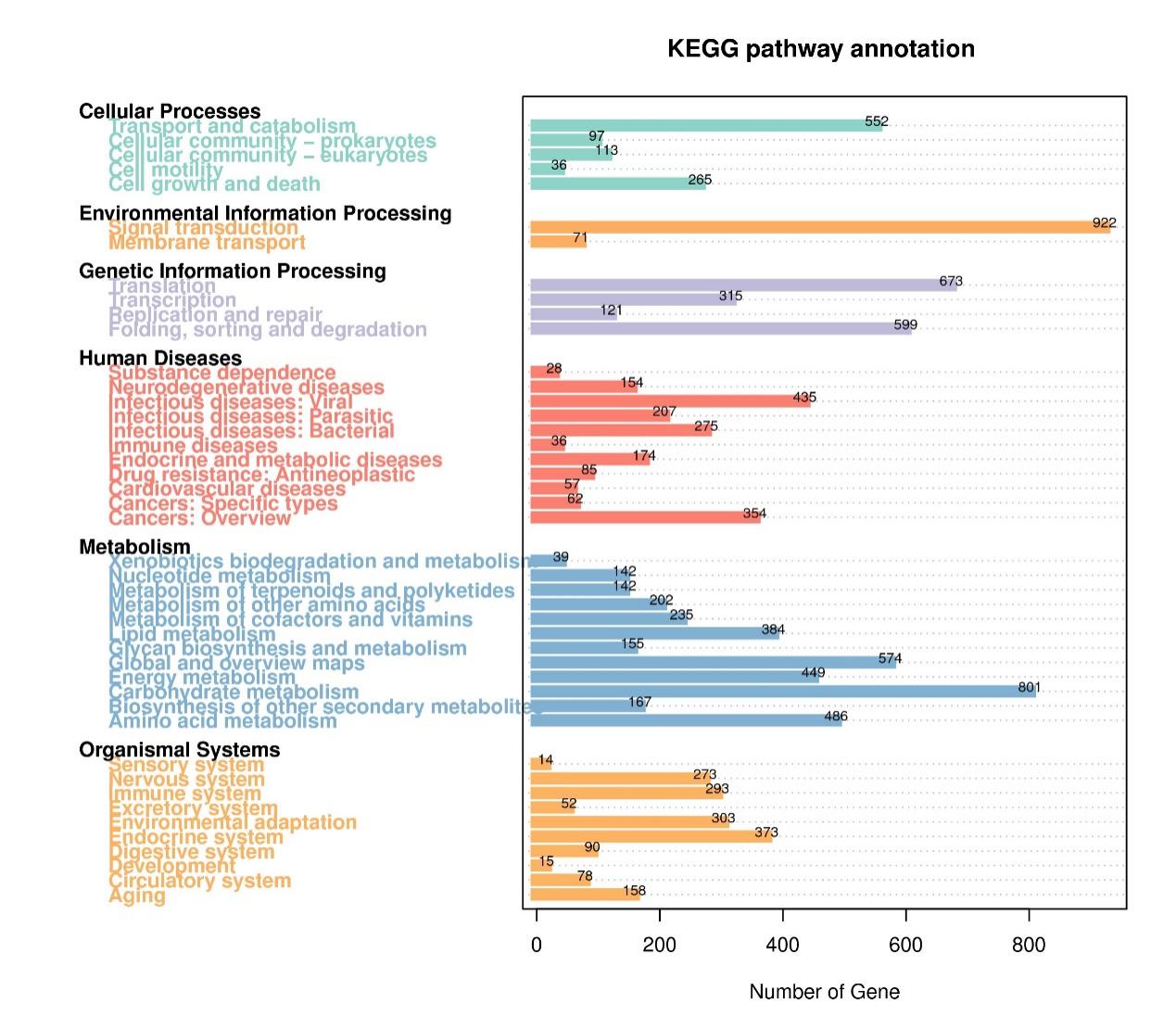


Supplementary Fig. S8 KEGG enrichment analyses of the genes of dandelion.


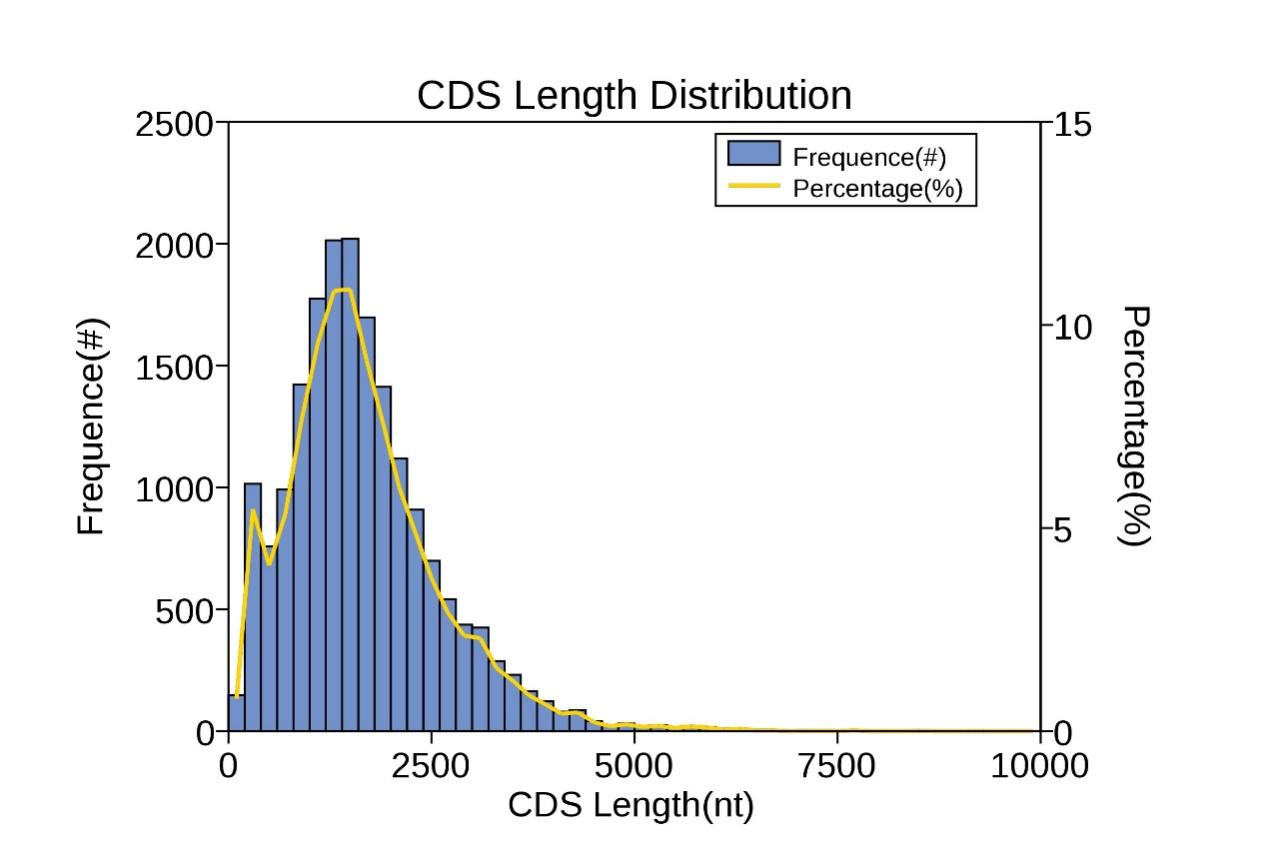


Supplementary Fig. S9 Statistics on the number of different length of predicted CDS.


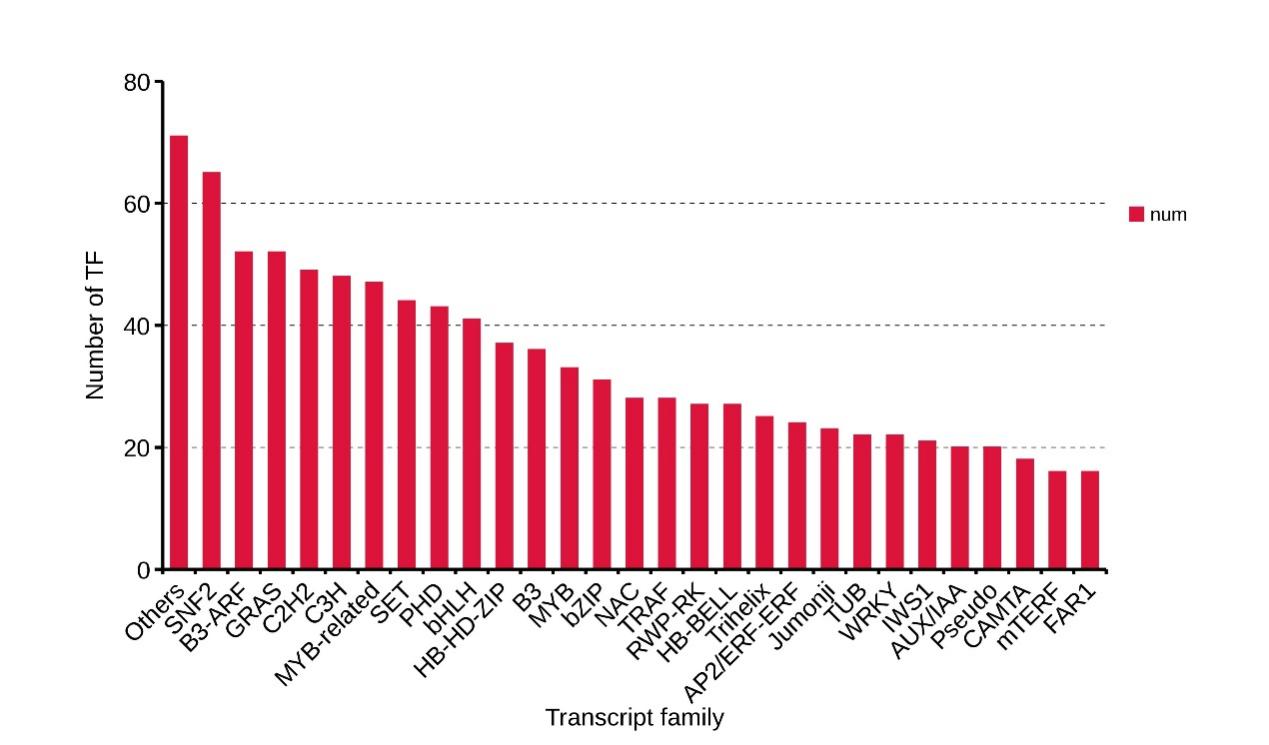


Supplementary Fig. S10 Transcription factor family classification.
